# Changes to inflammatory markers during 5 years of viral suppression and during viral blips in people with HIV initiating different integrase inhibitor based regimens

**DOI:** 10.3389/fimmu.2024.1488799

**Published:** 2024-11-12

**Authors:** Nicholas T. Funderburg, Susie S. Y. Huang, Calvin Cohen, Kate Ailstock, Morgan Cummings, Jean C. Lee, Brenda Ng, Kirsten White, Jeffrey J. Wallin, Bryan Downie, Grace A. McComsey

**Affiliations:** ^1^ School of Health and Rehabilitation Sciences, Division of Medical Laboratory Science, The Ohio State University, Columbus, OH, United States; ^2^ Gilead Sciences Inc., Foster City, CA, United States; ^3^ Case Western Reserve University, University Hospitals of Cleveland, Cleveland, OH, United States

**Keywords:** HIV-1, antiretroviral therapy, inflammation, intermittent viremia, monocyte activation

## Abstract

**Background:**

Heightened levels of inflammatory markers are linked to increased morbidity/mortality in people with HIV (PWH) and often remain elevated after virologic suppression by antiretroviral therapy (ART). As new combinations of ART become available, an evaluation of their effects on immune activation and inflammation is warranted. Additionally, it remains unknown whether transient increases in viral load (“blips”) during ART are associated with increases in inflammation.

**Methods:**

We utilized cryopreserved samples from treatment-naïve PWH enrolled in two Phase 3 clinical trials investigating the efficacy and safety of bictegravir, emtricitabine and tenofovir alafenamide (B/F/TAF) or dolutegravir, abacavir, and lamivudine (DTG/ABC/3TC) or DTG + F/TAF over a 5-year window (GS-US-380-1489/1490). At week 144, participants were offered the option to switch to open label B/F/TAF for an additional 96 weeks. We measured levels of interleukin-6 (IL-6), C-reactive protein (hsCRP), D-dimer, soluble CD14 (sCD14), and tumor necrosis factor-α receptor 1 (TNFR1) from available baseline, week 24, 48, 144, and 240 samples (B/F/TAF, N=123; DTG/ABC/3TC, N=62; DTG+F/TAF, N=58). Additional samples from PWH who experienced a viral blip (n=44, defined as a single HIV-1 RNA >50c/mL) were also analyzed and paired with the most recent available suppressed sample before the blip. Longitudinal biomarker changes were assessed using a constrained mixed effects linear regression model adjusting for covariates.

**Results:**

Baseline demographics and selected laboratory characteristics were similar across groups. Levels of D-dimer, sCD14, and TNFR1 decreased significantly from baseline in all treatment arms, with no significant differences between arms at any timepoint. Biomarker levels also remained stable following ART-switch at week 144. No significant changes in hsCRP or IL-6 were observed versus baseline in any arm at any timepoint. A significant association was observed between sCD14 and increasing viral load (p=0.022) in viral blips; D-dimer also increased with blips in the B/F/TAF arm.

**Conclusions:**

Viral suppression was associated with reductions in most inflammatory markers in PWH, with no significant differences among the three ART regimens during the 144-week randomized period. These decreases were sustained after the open label switch to B/F/TAF. Viral blips were associated with increases in monocyte activation (sCD14). Further analysis is needed to confirm these findings and determine the potential impact on clinical outcomes.

## Introduction

Treatment with combination antiretroviral therapy (ART) increases the expected lifespan of people with HIV (PWH), however, even when ART is successful at lowering HIV levels below the standard assay limit of detection, elevated levels of inflammation, immune activation, and hypercoagulation may persist (1–4). Chronic inflammation likely contributes to an increased risk of several comorbid conditions in PWH, including cardiovascular disease, insulin resistance and type II diabetes, osteoporosis, neurocognitive dysfunction, and frailty (2). Multiple mechanisms may contribute to inflammation, immune activation, and hypercoagulation in PWH, including low level viral replication, co-pathogens, microbial translocation, ART-related toxicities, oxidative stress, and altered lipid profiles (2). The Strategies for Management of Anti-Retroviral Therapy (SMART) trial explored the consequences of continuous viral suppression by ART versus limiting ART exposure in PWH. Viral suppression was beneficial within both treatment arms, but continuous inhibition had superior benefit over intermittent suppression for AIDS- and non-AIDS events and mortality (5, 6). Plasma levels of interleukin (IL)-6, high-sensitivity C-reactive protein (hsCRP), D-dimer, and soluble CD14 (sCD14) were independent predictors of morbidity and mortality in this study (5–7). Other inflammatory markers, including tumor necrosis factor receptors 1 and 2 (TNFR1 and TNFR2) (8–10) are also predictive of morbidity and mortality in PWH. Further, combinations of biomarkers (e.g. IL-6 and D-dimer and/or IL-6 and TNFR1) (11, 12) may have enhanced predictive value for adverse health outcomes compared to levels of individual proteins.

Long lived cells infected with HIV-1, including memory CD4+ T cells and macrophages, may contribute to persistent production of HIV-1 associated RNA molecules and proteins (13, 14). Activation of these latently infected cells may contribute to measurable levels of HIV-1 viremia, typically >50 copies/mL, often referred to as viral “blips.” These blips could result from an interruption in suppressive ART or through mechanisms that induce viral replication. The levels to which persistent HIV-1 replication products and/or virologic blips contribute to chronic inflammation in PWH have not been thoroughly investigated (13, 14).

As new ART drugs become available, assessment of the virologic, immunologic, and metabolic effects of these drugs in well-controlled clinical trials is needed. Improved ART combinations should rapidly and consistently decrease plasma HIV-1 levels, have minimal effects on metabolic profiles, and improve immune cell function while minimizing persistent immune activation and inflammation in PWH. Studies GS-US-380-1489,1490 evaluated the efficacy and safety of currently recommended INSTI plus two NRTI ART combinations bictegravir (B), emtricitabine (F), tenofovir alafenamide (TAF), compared to dolutegravir (DTG), F and TAF, or DTG, abacavir (ABC) and lamivudine (3TC) (15–18) in treatment naïve PWH. These studies demonstrated that B/F/TAF is a well-tolerated and effective ART regimen over a duration of 5 years, with rapid suppression of viral replication and minimal effects on lipid profiles, bone density, and kidney function (19). Here, using a subset of participants from these parent studies, we explored the longitudinal effects of these ART regimens on five soluble markers of inflammation and immune activation that have been previously linked to morbidity and mortality in PWH (4–8, 11).

## Materials and methods

### Study design and sample selection

We utilized cryopreserved samples in a convenience sampling of US participants from the two Phase 3 clinical studies that enrolled treatment-naïve PWH and randomized them to receive either bictegravir, emtricitabine and tenofovir alafenamide (B/F/TAF) or dolutegravir, abacavir, and lamivudine (DTG/ABC/3TC) in Study 1489 or DTG + F/TAF in Study 1490 (15–18). All participants were switched to B/F/TAF at week 144 for a 96-week open label extension (OLE). Samples from baseline, week 24, 48, 144, and 240, were used from participants who had consistently suppressed viremia (HIV-1 RNA <50 copies/mL at week 24). Samples from participants with baseline viral loads (VL) of HIV-1 RNA >5log were over selected to assure significant decreases in markers of immune activation following ART initiation (N=50 in each arm). As well, all samples from eligible female participants were included for the analysis to be more representative of sex as well as to explore sex as a biological variable. Participants who failed to complete the trial to week 240 (Wk 96 OLE), had co-infection with hepatitis C virus (HCV), or who developed an underlying comorbid condition, such as COVID diagnosis, cancer diagnosis and statin initiation, that may complicate their inflammatory profiles were excluded. Finally, samples were selected to balance selected patient characteristics (age, sex, CD4+ T cell count at ART initiation), across treatment arms which may play a role in immune profiles.

### Measurement of plasma biomarkers

Plasma samples were identified and shipped on dry ice for measurement of markers of immune activation and inflammation using enzyme-linked immunosorbent assays (ELISA) at Ohio State University (Columbus, Ohio, USA). Measured biomarkers included soluble CD14 (sCD14), high-sensitivity C-reactive protein (hsCRP), interleukin-6 (IL-6), tumor necrosis factor receptor 1 (TNF-R1) (R&D Systems, Minneapolis, Minnesota, USA) and D-dimer (Diagnostica Stago, Parsippany, New Jersey, USA). To the extent possible, assays were performed by the same person each time, limiting operator variability and each assay plate was from the same manufacturer and lot. In brief, samples were diluted using the dilution factors that have been identified during our previous studies (20–24). Standard curves were run in duplicate per plate, and samples were re-run at a different dilution if values were out of range or if the duplicated sample values were not within manufacturer’s assay range. A threshold of CV<20% for repeats was applied to all technical duplicates. Each sample was thawed once and used on all plates run on that day.

### Statistical analyses

Longitudinal biomarker changes were assessed from log-transformed values, using a constrained mixed-effects linear regression model (LMEM; R package “lme4”) with time as categorical values and adjusting for covariates (sex and baseline viral load). Associations between inflammation biomarkers and viral load were tested with a B-spline LMEM, with the R packages “lme4” and “spline”, adjusting for baseline viral load, treatment arm, and time. Baseline associations between CD4+ T cell count/CD4+ percentage and biomarker levels were performed using Spearman’s Correlation. Associations of longitudinal changes in CD4+ T cell count/CD4+ percentage and biomarker levels were tested with LEME using “lme4”, adjusting for changes of viral load from baseline, treatment arm, and time. Significance was determined by a false discover rate (FDR) < 0.05 unless otherwise stated. Nonparametric tests (Wilcoxon Signed Rank Test or Wilcoxon Rank Sum Test and Kruskal Wallis Test) were used to compare between groups where appropriate and as indicated. All analyses were performed in R (version 4.2.1).

## Results

### Characteristics of study participants

**Figure 1:**
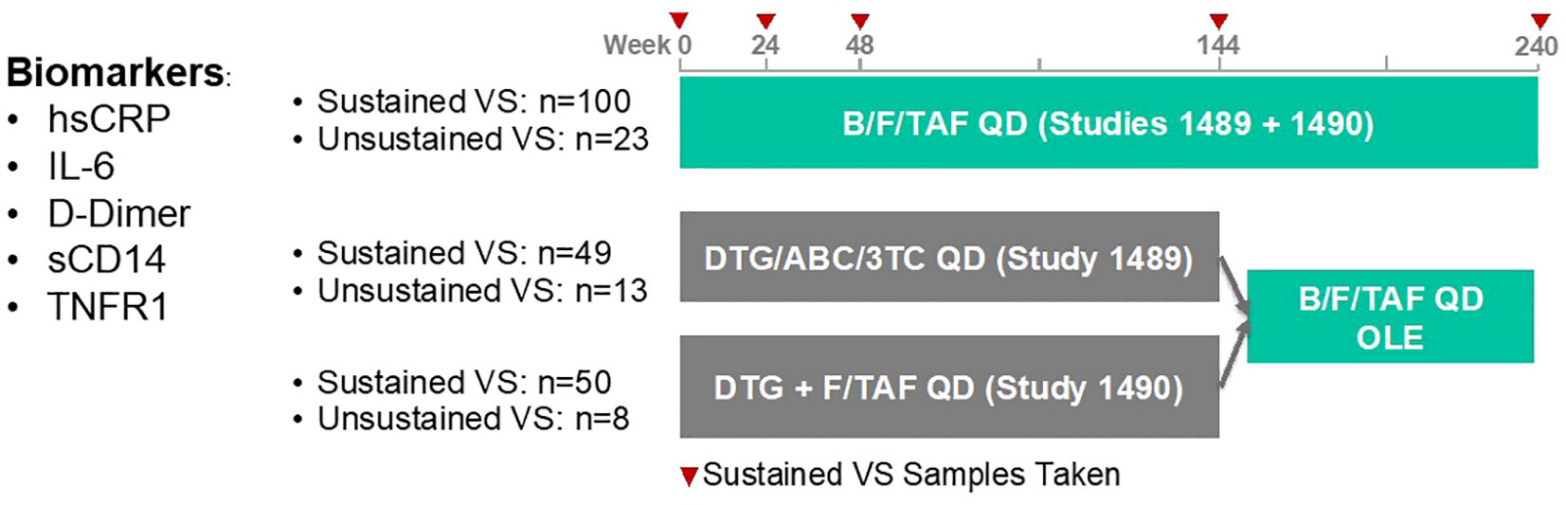
GS-US-380-1489/90 clinical study design and plan for retrospective biomarker analysis. We utilized cryopreserved samples from a sampling of treatment-naïve PWH in the United States who are enrolled in two Phase 3 clinical studies investigating the efficacy and safety of B/F/TAF, DTG/ABC/3TC and DTG+F/TAF over a 5-year window. Samples from sustained VS participants with baseline viral loads (VL) > 100,000 cp/mL were over selected. Participants with an underlying comorbid condition that complicated their inflammatory profiles were excluded.

We utilized cryopreserved samples from a sampling of treatment-naïve PWH enrolled in two Phase 3 clinical studies investigating the efficacy and safety of B/F/TAF, DTG/ABC/3TC and DTG+F/TAF over a 5-year window (GS-US-380-1489/1490, **Figure 1**). At wk 144, participants were offered a switch to open label B/F/TAF. In general, selected participant demographic and clinical characteristics were balanced across treatment arms (**Table 1**). Participants in the DTG+F/TAF arm were observed to have lower baseline CD4+ T cell counts compared to baseline CD4+ T cell counts in the other two treatment arms (p=0.01), however viral loads were generally balanced (p=0.34).

**Table 1:** Demographic and clinical information on study participants.

Median [Q1,Q3]	B/F/TAF N=100	DTG/ABC/3TC N=49	DTG + F/TAF N=50	P value
Age (yrs)	29 (25, 39)	31 (24, 40)	34 (26, 49)	0.61
Sex (M/F)	86/14	41/8	44/6	0.83
BMI	25.6 (23.2, 29.9)	24.57 (23.2, 26.9)	24.19 (21.5, 28.3)	0.04
eGFR (mL/min)	130.1 (108.9, 152.8)	127.1 (112.5, 140.5)	118.2 (103.6, 146.1)	0.66
Viral Load (copies/mL)	37650 (16500, 111250)	33800 (7770, 139000)	48900 (11095, 170250)	0.39
CD4 (%)	21.8 (16.9, 27.9)	23.6 (19.6, 31.1)	19.05 (14.5, 24.6)	0.33
CD4 (cells/uL)	411 (260, 517)	451 (250, 583)	342 (199, 557)	0.01
Fasting Cholesterol (mg/dL)	155.5 (119.3, 182.8)	153 (138, 182)	161 (122.8, 195.3)	0.36
Fasting HDL Cholesterol (mg/dL)	49 (33.5, 60)	44.5 (36.5, 55.5)	29 (19.5, 64)	0.32
Fasting Total Cholesterol to HDL Ratio	3.7 (3.03, 4.28)	3.49 (3.04, 4.38)	3.92 (3.1, 4.72)	0.39
Fasting Triglycerides (mg/dL)	97 (71, 145)	93.5 (63, 152.8)	138 (114.5, 179)	0.71

P values were obtained from Kruskal Wallis Test among treatment arms.

### Changes in inflammatory markers following ART initiation

**Figure 2:**
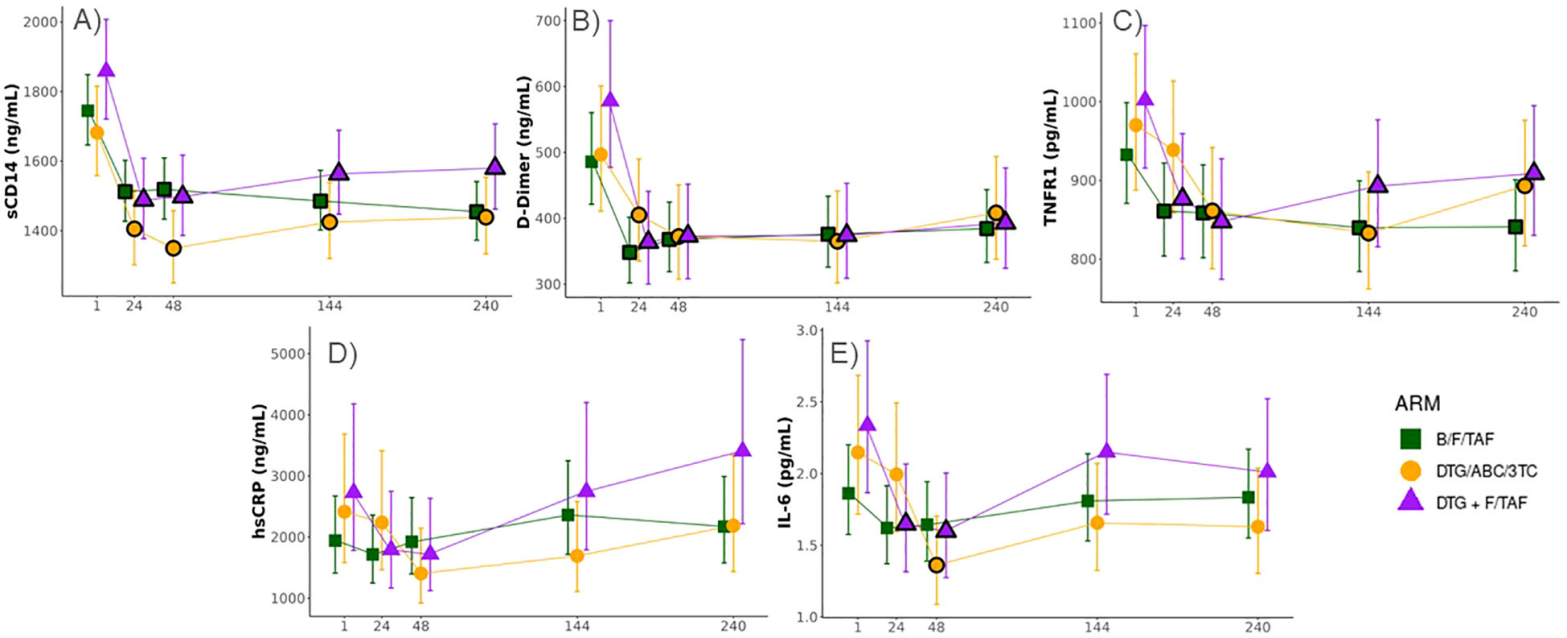
Significant decreases in biomarkers observed across all ART treatment arms in PWH. Longitudinal changes in serum biomarkers associated with inflammation (Least-square means ± 95%CI; statistical differences against baseline and between Week 144 and Week 240 were determined from log2 transformed data and a constrained mixed effects linear regression model adjusted for sex and baseline viral load). Levels of **(A)** sCD14, **(B)** D-Dimer, **(C)** TNFR1, **(D)** hsCRP, and **(E)** IL-6 were measured by enzyme linked immunosorbent assay (ELISA). Black borders on the symbols denote a significant difference from baseline at a false discovery rate (FDR) < 0.05.

Serum levels of IL-6, hsCRP, D-dimer, sCD14, and TNFR1 were measured by ELISA in participants at baseline and selected time points. Levels of all biomarkers at baseline tended to be highest in the DTG+F/TAF group. Significant decreases (FDR<0.05) in levels of sCD14, D-dimer and TNFR1 from baseline were observed for each study arm and reductions were largely maintained throughout the randomized treatment timepoints as well as the open label extension (OLE, **Figures 2A–C**). IL-6 and hsCRP showed a tendency to decrease from baseline by week 48 across study arms (**Figures 2D, E**) but did not generally exhibit significant differences after multiple testing correction. No significant differences were observed in biomarker changes between treatment arms. As expected, most inflammatory markers were significantly higher (p<0.03 for all but hsCRP) at baseline in participants with high VLs (>100,000 copies/mL, N=64) compared to levels in participants who had lower VLs (<100,000 copies/mL, N=132) (**Table 2**). Nevertheless, changes in inflammatory markers were similar in participants with high and low VLs at baseline, with the magnitude of biomarker decreases largest in the high VL group (**Supplementary Figure S1**, **S2**).

**Table 2:** Baseline biomarker levels were higher in participants with high baseline viral load.

Marker	VL > 100,000 cp/mL (n=64)	VL ≤ 100, 000 cp/mL (n=132)	padj
hsCRP (ng/mL)	1516 (615-4670)	1352 (642-3767)	0.57
IL-6 (pg/mL)	2 (1.2-3)	1.5 (0.9-3)	0.03
D-Dimer (ng/mL)	423 (304-792)	356 (249-571)	0.03
sCD14 (ng/mL)	1947 (1557-2340)	1688 (1411-1918)	<0.01
TNFR1 (pg/mL)	1032 (823-1287)	877 (750-1057)	<0.01

Data are shown as Median (IQR) and differences were analyzed using a two sided Wilcoxon Rank Test.

**Figure 3:**
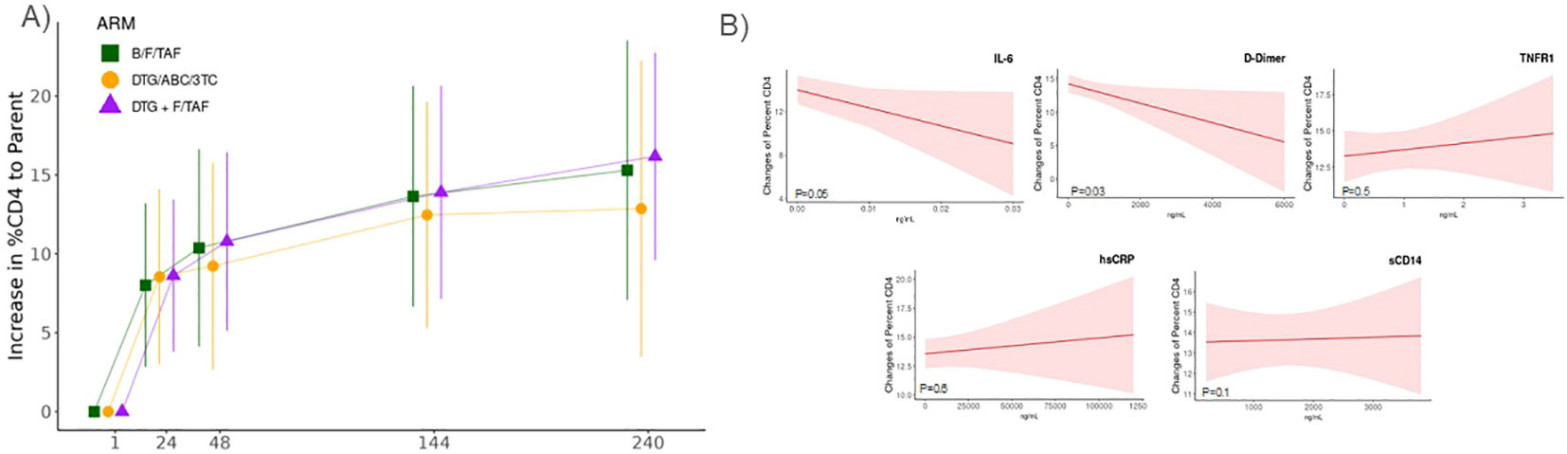
Changes in inflammatory markers were associated with increases in CD4+ T cell percentages. Association of changes in CD4+ T cell percentages and inflammatory biomarker levels derived from a linear mixed effect model adjusting for difference to baseline viral load, treatment arm, and time. **(A)** CD4+ percentage increases overtime in each treatment arm. **(B)** Changes in CD4+ T cell percentages (least-square means ± 95%CI) in relation to levels IL-6, D-Dimer, TNFR1, sCD14, or hsCRP that were measured by enzyme linked immunosorbent assay (ELISA).

We also sought to investigate relative longitudinal changes in these inflammatory biomarkers with changes in CD4+ T cell count and CD4+ percentage. Correlations were observed for all biomarkers and CD4+ T cell count and %CD4+ T-cells at baseline (**Supplementary Table S1**). Decreases of inflammatory biomarkers were inversely associated with increases in CD4+ T cell counts and %CD4+ T cells over time (**Figures 3A, B**, **Supplementary Table S2A, B**), independent of treatment arm and adjusted for changes in viral load. The strongest relationships were observed for IL-6 (p= 0.038 and 0.05, respectively), with a 1% decrease in IL-6 corresponding to a 68.03 cell increase in CD4+ T cells/µL (**Supplementary Table S2A**) and a 1.65% increase in %CD4 T-cells (**Supplementary Table S2B**). A similar pattern was observed between changes in CD4 T cell counts and hsCRP (p=0.04) and sCD14 (p<0.01) and changes in CD4% and D-Dimer (p=0.03), although the effect magnitudes on CD4 counts and CD4% were substantially smaller (**Figure 3** and **Supplementary Table S2A, B**).

### Changes in inflammatory markers associated with viral blips

**Figure 4:**
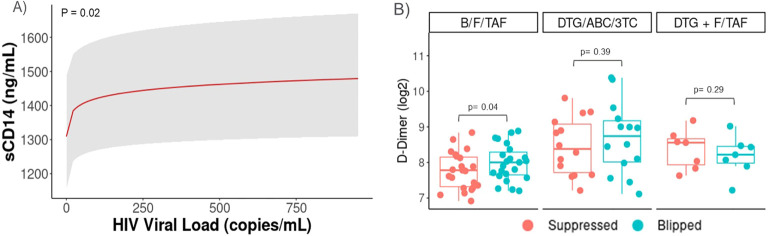
Transient viral elevations (Blips) were associated with increases in sCD14 and D-dimer levels. Data from PWH who experienced a viral blip (n=44, defined as single 50c/mL> HIV-1 RNA < 1000c/mL) during treatment were also analyzed and paired with the most recent suppressed sample before the blip. Participants with viral blips in each arm were included (B/F/TAF, n=23, DTG/ABC/3TC, n=13, and DTG+F/TAF, n=8). Associations between inflammation biomarkers and viral load were tested with a B-spline LMEM adjusting for baseline viral load, treatment arm, and time. Least-squares means and confidence intervals shown for **(A)** sCD14 and median (IQR) shown for **(B)** D-Dimer, with p-values tested by Wilcoxon Signed Rank Test.

Next, we focused on a subgroup of participants from across all arms who experienced a period of intermittent viremia, or HIV-1 viral blips. A subset of samples from PWH who experienced a viral blip (n=44, defined as single 50 c/mL > HIV-1 RNA < 1000 c/mL) during treatment were analyzed and paired with the most recent suppressed sample before the blip. Associations between inflammation biomarkers and viral load were tested with a B-spline LMEM adjusting for baseline viral load, treatment arm, and time. A significant positive association (p=0.02) was observed between increasing viral load and sCD14 during blips (**Figure 4A**). Wilcoxon Signed Rank Test was then used to compare biomarker levels between paired control and blips samples. Levels of D-dimer were found to be significantly higher (p=0.04) in those with transient increases in viral load in the B/F/TAF arm (**Figure 4B**), but statistically significant differences were not detected in participants in the other arms.

## Discussion

Persistent elevation in levels of markers of immune activation and inflammation are associated with morbidity and mortality in PWH, including the increased risk of cardiometabolic disease that has been reported in this population (2, 25). There are likely multiple potential mediators of chronic immune activation in this population, including low level HIV-1 replication, co-infections, microbial translocation, and alterations in proinflammatory lipid profiles (2, 25). Many of these mediators can drive activation of innate (i.e., monocytes, macrophages, natural killer cells) and adaptive (i.e., T and B lymphocytes) immune cells, triggering intracellular signaling cascades (e.g., NFκB, the inflammasome) that modulate gene and protein expression and increase production of pro-inflammatory molecules. Several strategies to reduce chronic immune activation in PWH have been explored, including the optimization of ART regimens. Here, in a comparison of three different ART regimens, those containing DTG, TAF and/or BIC, we report that each of these regimens reduce levels of several inflammatory markers (sCD14, TNFR1, D-dimer) that have been associated with morbidity and mortality in PWH (5, 7, 8) and that reductions in these molecules persisted for over 5 years.

Newer ART drugs with improved profiles for viral suppression and reduced toxicity are being developed. Studies with these new agents have also focused on comparisons of the speed, magnitude, and durability of reductions in immune activation and inflammation between drugs and drug regimens (1). We have previously reported that changes in immune activation markers were not different in ART- naïve PWH initiating a regimen with either tenofovir disoproxil fumarate (TDF) or TAF (22). As newer reverse transcriptase inhibitors become available, similar studies should be performed.

Integrase inhibitors may decrease inflammation and immune activation more than other antiretroviral classes. Mechanisms for this difference could include findings that integrase inhibitors may be more lipid neutral (26) and concentrate at higher levels in enterocytes (27), resulting in improved gut barrier integrity and reduced translocation of microbial products from the gut lumen. Reducing microbial translocation and limiting adverse lipid effects may improve chronic inflammation in PWH. We have reported rapid declines in markers of immune activation (including IL-6, hsCRP, D-dimer, TNFR1, and sCD14) in ART naïve participants initiating a raltegravir (RAL) based regimen (20). In a study where ART- naïve participants were randomized to elvitegravir/cobicistat/tenofovir disoproxil fumarate/emtricitabine (EVG/c/TDF/FTC) or efavirenz/TDF/FTC (EFV/TDF/FTC) changes in inflammatory markers were compared in a subset of participants with suppressed viremia at week 48 (28, 29). Initiation of the integrase inhibitor-based regimen resulted in greater and more rapid declines in hsCRP, sCD14, and the vascular inflammation marker Lp-PLA2; the magnitude of changes in other markers (IL-6, TNFR1, and sCD163) was similar between arms. In ACTG 5260s, where ART-naïve participants were randomized to receive TDF/FTC plus open-label RAL, atazanavir/ritonavir (ATV/r), or darunavir/ritonavir (DRV/r) (26) the changes in inflammatory markers across groups were inconsistent; hsCRP decreased with ATV/r and RAL by 96 weeks; IL-6 decreased with RAL, but not with ATV/r and DRV/r; D-dimer decreased with ATV/r and DRV/r, but was unchanged with RAL; markers of T cell activation and sCD163, but not sCD14, decreased in all groups (30). In the SPIRAL study, where participants were switched from a boosted protease inhibitor (PI) to RAL or maintained their boosted PI regimen, switching to RAL resulted in greater decreases in multiple inflammatory markers, including IL-6, hsCRP, and D-dimer (31). Further studies are needed as innovative drugs emerge, including the relatively newer integrase inhibitors (e.g., DTG, BIC, cabotegravir), to determine if certain integrase inhibitors may be more effective at reducing immune activation than others. In this study, we did not find a significant difference between the longitudinal changes in inflammatory marker levels when comparing participants receiving DTG to those receiving BIC. We cannot discount the small sample size of our study and the potential small effects size of the treatment arm leading to low power. The current study was also not designed to test whether integrase-based regimens are more effective at reducing inflammation compared to other regimens, but as noted above, this has been and can continue to be explored in future studies.

While no significant changes in IL-6 levels from baseline were observed, a decrease in the levels of this pro-inflammatory cytokine may have important consequences for overall immune health. Here, we report a significant relationship among increasing CD4+ T cell counts and %CD4+ T cells and decreasing levels of IL-6 among participants. Pro-inflammatory cytokines, including IL-6 and IL-1β, can drive CD4+ T cell proliferation, dysfunction, and death (32, 33). In a recent study in suppressed PWH on ART, testing the blockade of the biological effects of IL-6 by tocilizumab, multiple markers of immune activation and CD4+ T cell cycling and PD-1 expression were decreased, reducing inflammation and potentially improving CD4+ T cell function (23). In our study, we also found a relationship between decreases in levels of TNFR1 and %CD4+ T cell increases, but the magnitude of this effect was much smaller than that of IL-6, highlighting the potential biological significance of decreasing IL-6 in PWH.

Persistent low-level HIV-1 viremia has been associated with chronic inflammation (13, 14) but the immunologic consequences of intermittent low-level viremia (i.e., virologic blips) are less clear. Here, we report a significant association between intermittent viremia and levels of sCD14 across participants in all arms and a relationship between blips and D-dimer levels in participants within the B/F/TAF arm. We have shown relationships among sCD14, monocyte expression of the procoagulant molecule tissue factor, and D-dimer levels in PWH with suppressed and unsuppressed viremia (21, 24). Further, HIV-1 could directly induce the expression of tissue factor on CD14^Dim^CD16^+^ monocytes (24) providing a plausible mechanism whereby increased levels of HIV-1 or viral replication products could drive monocyte activation and coagulation. Intermittent viremia may be caused by a number of factors, including inconsistent ART use, activation of a latently infected CD4+ T cell resulting in clonal expansion, or exacerbation of low level HIV-1 replication in tissue sites that becomes detectable in the circulation (34). We cannot determine the exact causes of virologic blips among participants in this study, but future studies designed to explore the causes and immunologic consequences of virologic blips are appropriate.

While this study provides insights into the relationships among changes in immune activation and CD4+ T cells during 5 years of ART treatment, and potential linkages between intermittent viremia and immune cell activation in PWH, there are limitations that should be considered. We did not find statistically significant changes in levels of IL-6 or hsCRP in study participants. This may be due, in part, because the baseline levels of these factors fell within the normal range (34, 36). Participants initiated ART with reasonably high levels of CD4+ T cell numbers, resulting in lower levels of IL-6 and hsCRP compared to other studies that show relationships between pre-ART levels of inflammation and morbidity/mortality (4, 5, 8). The relatively small sample size of participants who experienced a virologic blip and the indeterminant nature of the cause(s) of these blips should also be considered. Further, while all biomarkers measured in this study fell, to varying degrees, by week 48, many of the markers had slight upward trajectories at later timepoints. Week 240 for many participants fell during the onset of the COVID-19 pandemic and while we excluded for COVID diagnoses we cannot confirm whether or not all cases were captured, which could reasonably have influenced markers of immune activation and inflammation in this population. Even with these considerations, this work demonstrates the success of ART in blocking viral replication and improving immune profiles in PWH and suggests that viral blips may contribute to monocyte activation and coagulation in this population. Understanding the causes and immunologic consequences of persistent and intermittent HIV viremia should be explored further.

## Data Availability

The raw data supporting the conclusions of this article will be made available by the authors, without undue reservation.

## Supplementary material

Supplementary Figure 1 Levels of inflammatory markers overtime in participants with high baseline viral load. Longitudinal changes in serum biomarkers associated with inflammation (least-square means ± 95%CI; statistical differences against baseline and between Week 144 and Week 240 were determined from log2 transformed data and a constrained mixed effects linear regression model adjusted for baseline viral load and sex Levels of A) sCD14, B) D-Dimer, C) TNFR1, D) hsCRP, and E) IL-6 were measured by enzyme linked immunosorbent assay (ELISA). Black borders on the symbols denote a significant difference from baseline at a false discovery rate (FDR) < 0.05.

Supplementary Figure 2 Levels of inflammatory markers overtime in participants with low baseline viral load. Longitudinal changes in serum biomarkers associated with inflammation (Least-square means ± 95%CI; statistical differences against baseline and between Week 144 and Week 240 were determined from log2 transformed data and a constrained mixed effects linear regression model adjusted for baseline viral load and sex). Levels of A) sCD14, B) D-Dimer, C) TNFR1, D) hsCRP, and E) IL-6 were measured by enzyme linked immunosorbent assay (ELISA). Black borders on the symbols denote a significant difference from baseline at a false discovery rate (FDR) < 0.05.

Supplementary Table 1 Baseline biomarker levels are negatively associated with CD4% and CD4 Counts. Levels of plasma biomarkers were measured by enzyme linked immunosorbent assay (ELISA) and spearman correlations between baseline biomarkers and measures of CD4+ T cells are shown.

Supplementary Table 2 Increases in IL-6 from Baseline is associated with significant reduction in the increase in A) CD4+ T cell counts and B) percent CD4+ T cells. There were no differences between treatment arms.
