# Supplementary material for: Changes to inflammatory markers during 5 years of viral suppression and during viral blips in people with HIV initiating different integrase inhibitor based regimens
Source: Front Immunol. 2024 Nov 12;15:1488799. doi: 10.3389/fimmu.2024.1488799 (PMC11590120; doi:10.3389/fimmu.2024.1488799)
Supplement: Supplementary Figure 1 — Levels of inflammatory markers overtime in participants with high baseline viral load. Longitudinal changes in serum biomarkers associated with inflammation (least-square means ± 95%CI; statistical differences against baseline and between Week 144 and Week 240 were determined from log2 transformed data and a constrained mixed effects linear regression model adjusted for baseline viral load and sex Levels of A) sCD14, B) D-Dimer, C) TNFR1, D) hsCRP, and E) IL-6 were measured by enzyme linked immunosorbent assay (ELISA). Black borders on the symbols denote a significant difference from baseline at a false discovery rate (FDR) < 0.05. [file Presentation1.pptx]

## Slide 1
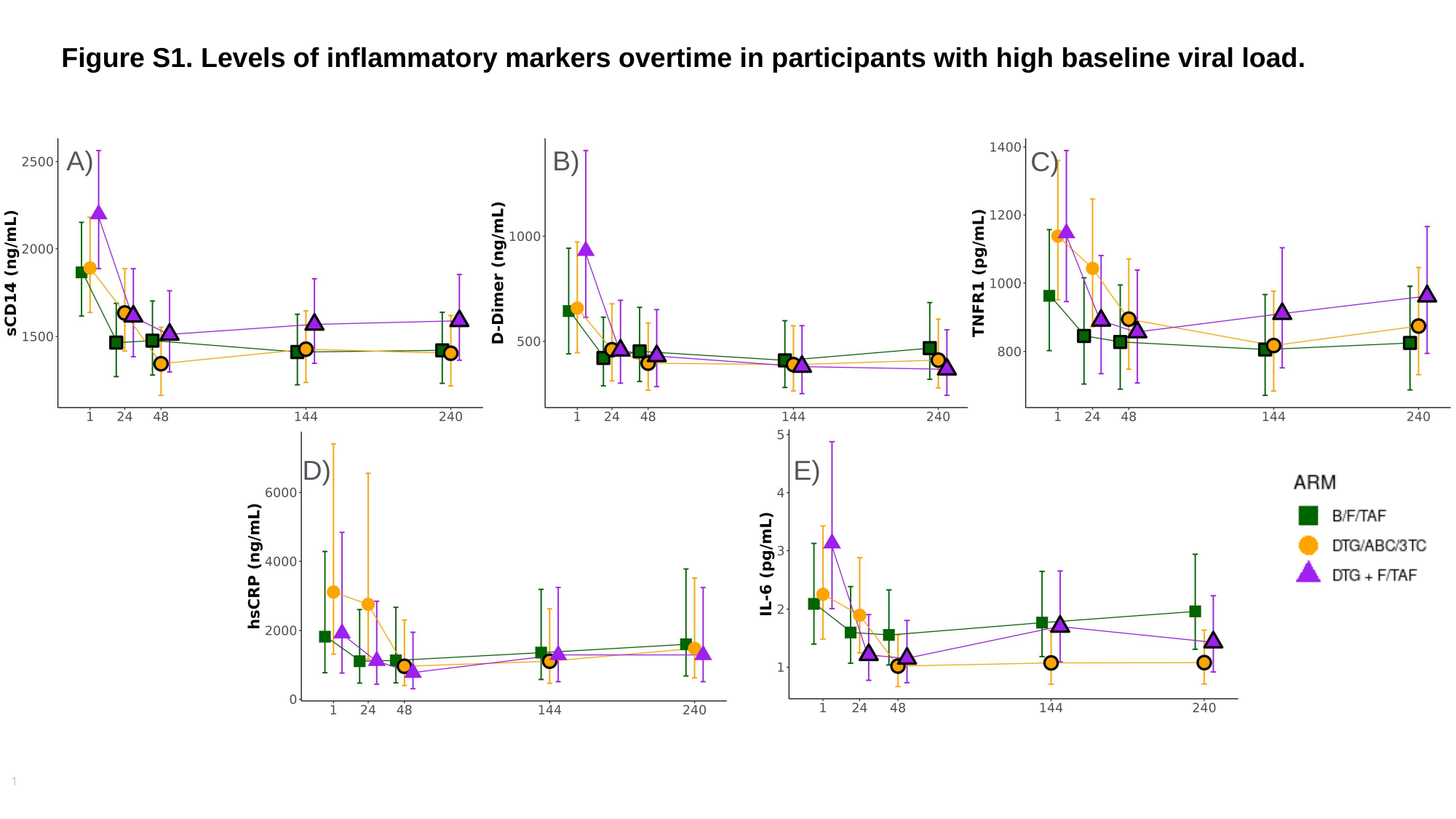

Figure S1. Levels of inflammatory markers overtime in participants with high baseline viral load.
A)
B)
C)
D)
E)
1

## Slide 2
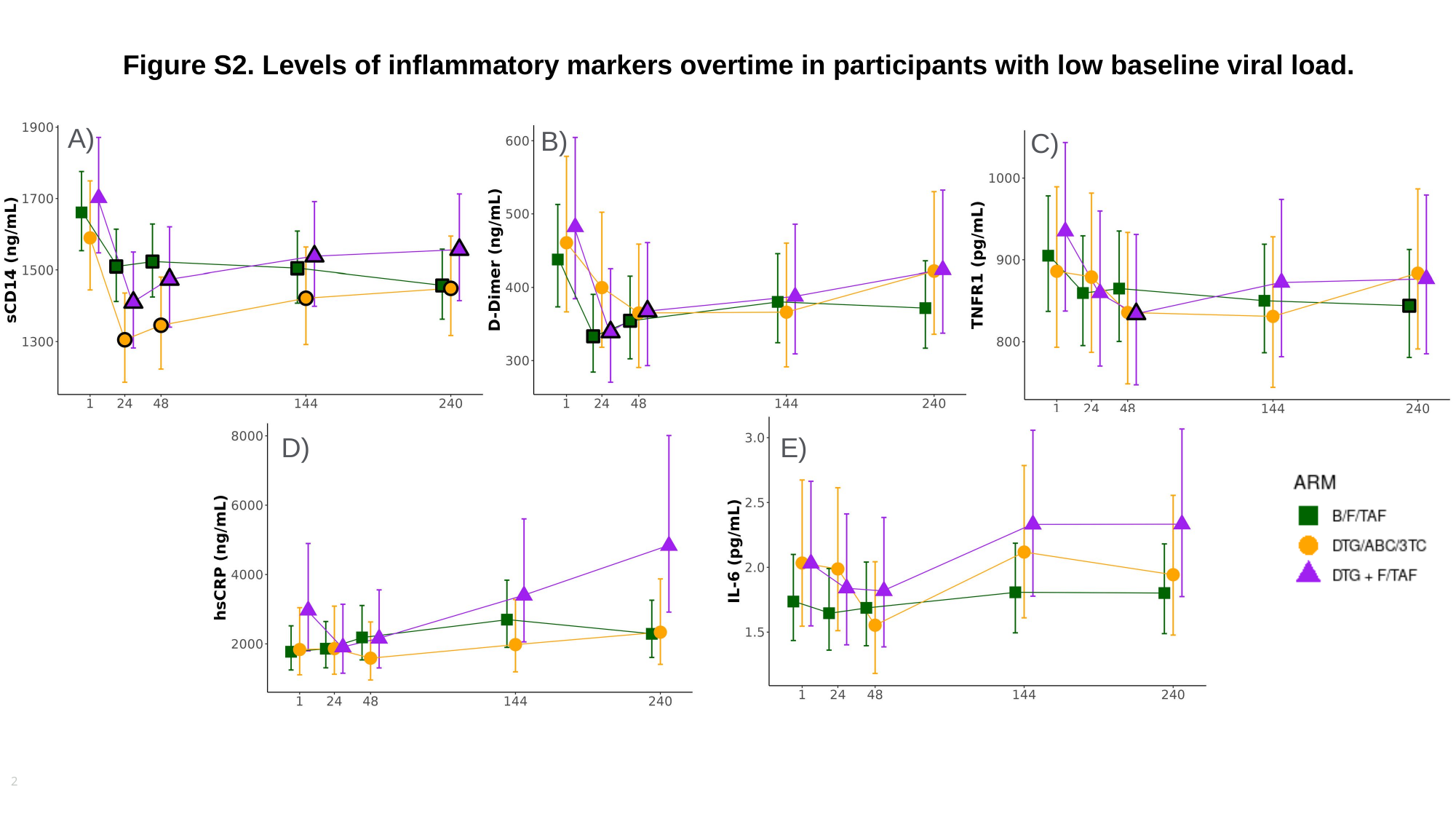

Figure S2. Levels of inflammatory markers overtime in participants with low baseline viral load.
A)
B)
C)
D)
E)
2

## Slide 3
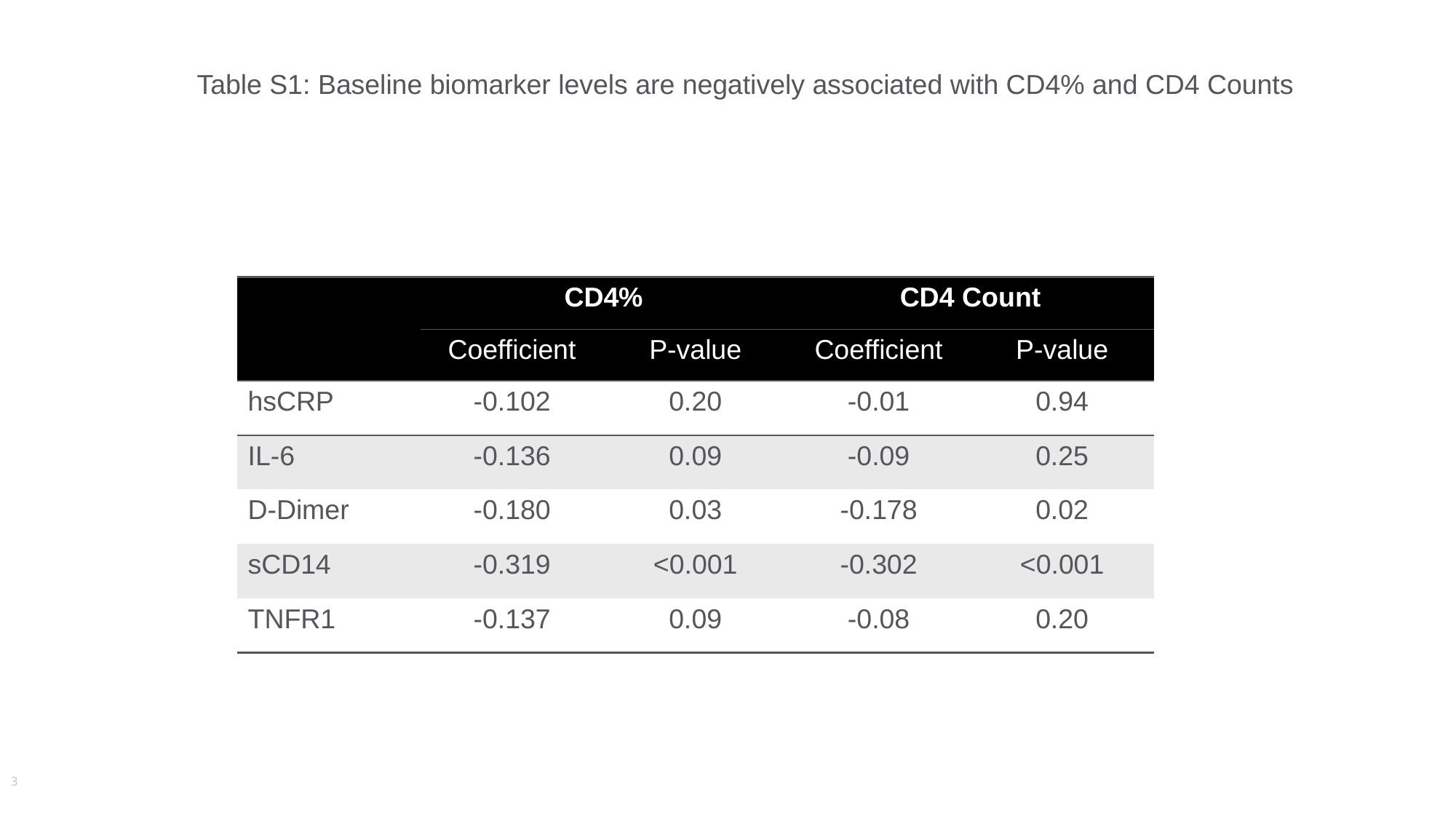

Table S1: Baseline biomarker levels are negatively associated with CD4% and CD4 Counts
| | CD4% | | CD4 Count | |
| --- | --- | --- | --- | --- |
| | Coefficient | P-value | Coefficient | P-value |
| hsCRP | -0.102 | 0.20 | -0.01 | 0.94 |
| IL-6 | -0.136 | 0.09 | -0.09 | 0.25 |
| D-Dimer | -0.180 | 0.03 | -0.178 | 0.02 |
| sCD14 | -0.319 | <0.001 | -0.302 | <0.001 |
| TNFR1 | -0.137 | 0.09 | -0.08 | 0.20 |
3

## Slide 4
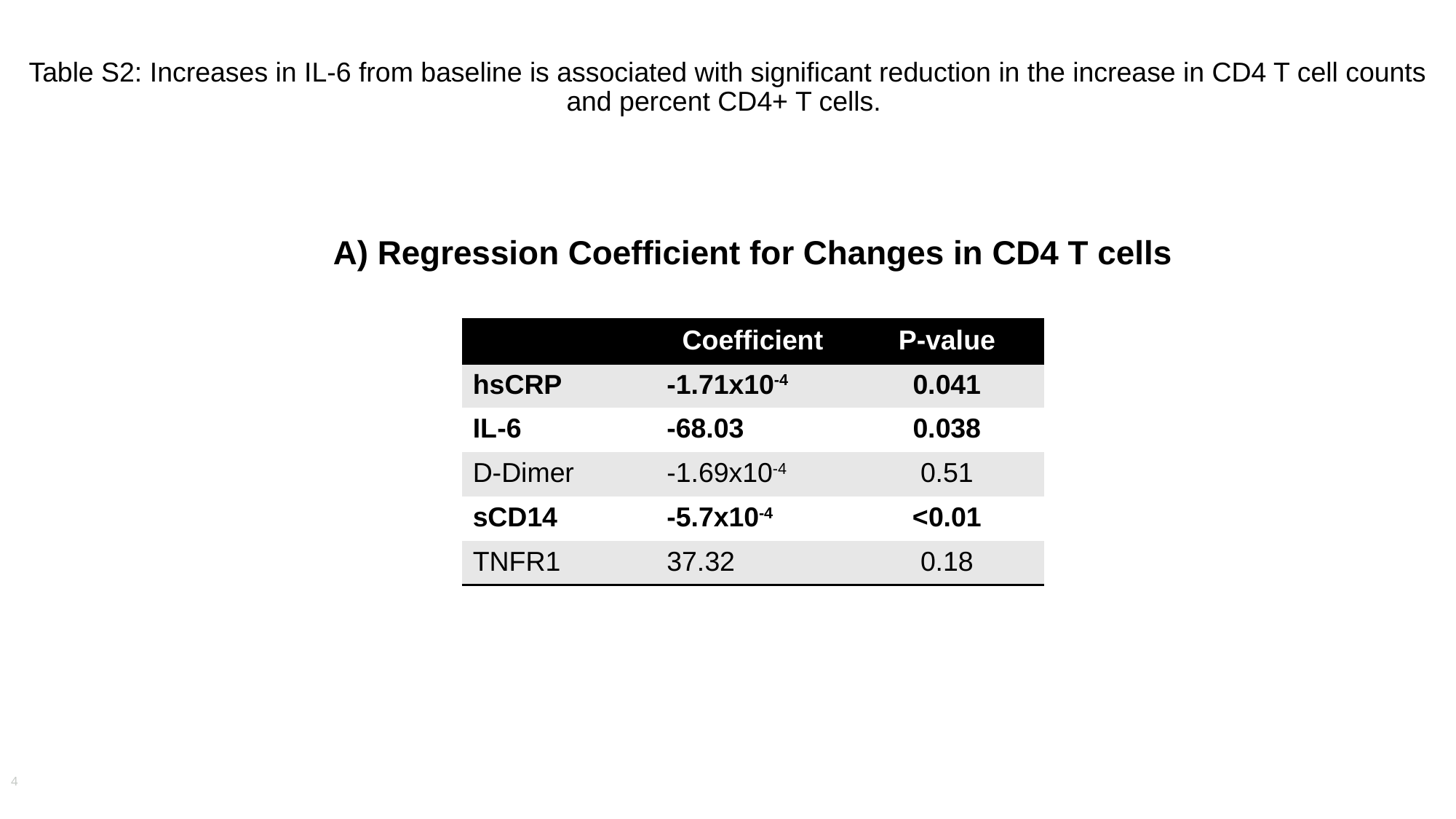

Table S2: Increases in IL-6 from baseline is associated with significant reduction in the increase in CD4 T cell counts and percent CD4+ T cells.
A) Regression Coefficient for Changes in CD4 T cells
| | Coefficient | P-value |
| --- | --- | --- |
| hsCRP | -1.71x10^-4^ | 0.041 |
| IL-6 | -68.03 | 0.038 |
| D-Dimer | -1.69x10^-4^ | 0.51 |
| sCD14 | -5.7x10^-4^ | <0.01 |
| TNFR1 | 37.32 | 0.18 |
4

## Slide 5
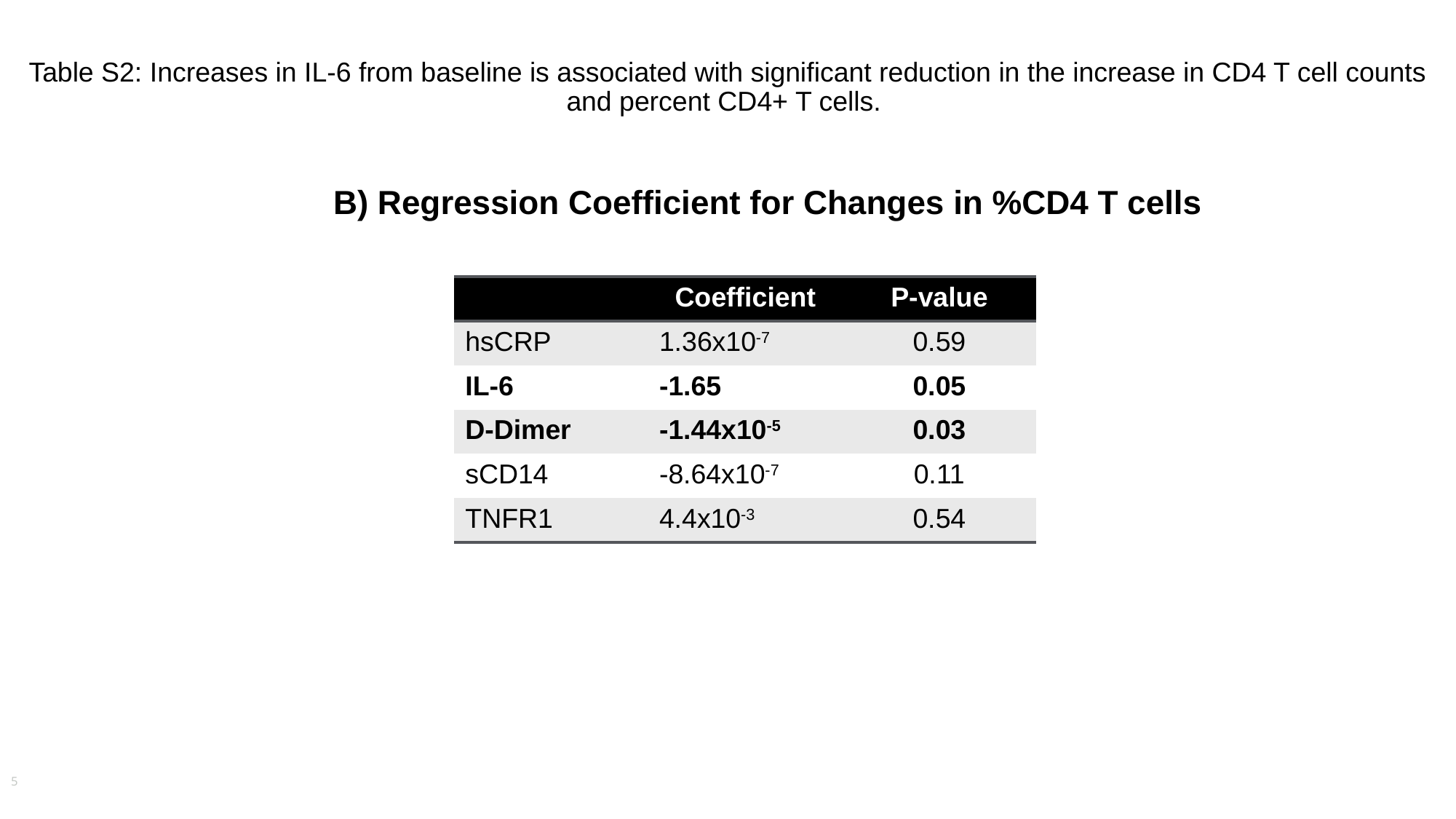

Table S2: Increases in IL-6 from baseline is associated with significant reduction in the increase in CD4 T cell counts and percent CD4+ T cells.
B) Regression Coefficient for Changes in %CD4 T cells
| | Coefficient | P-value |
| --- | --- | --- |
| hsCRP | 1.36x10^-7^ | 0.59 |
| IL-6 | -1.65 | 0.05 |
| D-Dimer | -1.44x10^-5^ | 0.03 |
| sCD14 | -8.64x10^-7^ | 0.11 |
| TNFR1 | 4.4x10^-3^ | 0.54 |
5
